# Review of the Narrow-Banded Hawkmoth, *Neogurelca montana* (Rothschild & Jordan, 1915) (Lepidoptera: Sphingidae) in China, with Morphological and Phylogenetic Analysis

**DOI:** 10.3390/insects14100818

**Published:** 2023-10-16

**Authors:** Zhen-Bang Xu, Ji-Bai He, Nan Yang, Ian J. Kitching, Shao-Ji Hu

**Affiliations:** 1 Yunnan Key Laboratory of International Rivers and Transboundary Eco-Security, Yunnan University, Kunming 650500, China; zhenbangxumm@gmail.com; 2 Institute of International Rivers and Eco-Security, Yunnan University, Kunming 650500, China; 3 Guangxi Institute of Botany, Chinses Academy of Sciences, Guilin 541006, China; 4 Independent Researcher, Chengdu 611139, China; hhh961201@163.com; 5 Beijing Baihuashan National Reserve, Beijing 102461, China; straybird@163.com; 6 Natural History Museum, Cromwell Road, London SW7 5BD, UK

**Keywords:** *Neogurelca*, DNA barcodes, molecular phylogeny, wing pattern, genital structure

## Abstract

**Simple Summary:**

The hawkmoth genus *Neogurelca* Hogenes & Treadaway, 1993 (Sphingidae: Macroglossinae: Macroglossini), comprises seven species, four of which are found in Asia and the other three in Central America. The members of this group in China are still poorly known, especially their distributions and taxonomy. We performed an analysis based on a 658 bp region of the COI mitochondrial gene, covering three species and including two populations of *N. montana* from Yunnan and Beijing, to resolve their phylogenetic positions. A morphological analysis of both sexes of the four Asian species was also undertaken. The phylogenetic analysis revealed the Beijing population of *N. montana* as a well-supported clade that is distinct from but close to the Yunnan population, which is consistent with the morphological differences between them. We describe the Beijing population of *N. montana* as a new subspecies, *Neogurelca montana taihangensis* **ssp. nov.**, based on our analysis. Additionally, we collected larvae of *N. montana* in Beijing and describe its life history and larval hosts and compare them with those of *N. himachala*.

**Abstract:**

*Neogurelca montana* (Rothschild & Jordan, 1915) is a species of the genus *Neogurelca* Hogenes & Treadaway, 1993, that was previously known from Sichuan, Yunnan, and Tibet, China. Recently, however, this species was also found in Beijing and Hebei. These populations differ from those in southwest China in body colour and the shape of the yellow patches of the hindwing—a paler body colour and triangular patches in the former and darker body colour and fan-like patches in the latter. Wing morphology, male and female genitalia, and molecular evidence (DNA barcodes) were analysed for the different localities of this species and three other *Neogurelca* species—*N. hyas*, *N. himachala*, and *N. masuriensis*. Our molecular data support the Beijing population of *montana* as a valid subspecies, which we describe as *N. montana taihangensis* **ssp. nov**. Wing and genital morphology confirm the molecular conclusions. We also collected larvae of the new subspecies in the Beijing suburbs and describe its life history and larval hosts and compare them with those of *N. himachala*.

## 1. Introduction

The hawkmoth genus *Neogurelca* Hogenes & Treadaway, 1993, comprises seven known species [1,2], including three species found in Central America, namely *N. sonorensis* (Clark, 1919), *N. mulleri* (Clark, 1923), and *N. serranoi* Haxaire, 2021, and four species in Asia, namely *N. hyas* (Walker, 1856), *N. masuriensis* (Butler, 1875), *N. himachala* (Butler, 1876), and *N. montana* (Rothschild & Jordan, 1915) [3,4,5,6,7,8]. The narrow-banded hawkmoth, *Neogurelca montana*, was first described by Rothschild and Jordan as *Gurelca montana*, based on four male syntypes deposited in the Natural History Museum London, U.K. (NHMUK). In the original description, the type locality was given only as “Tibet, without more definite locality”. However, at that time, the geographical term “Tibet” referred to a much wider region than the present province, consisting not only of present-day Tibet but also including the western part of Sichuan and northwestern part of Yunnan provinces [9,10]. Since its discovery, the distribution of *N. montana* has not been thoroughly discussed, nor has the female of this species been reported and described.

In 1922, Rudolf Emil Mell published a series of life-history records of hawkmoths in China [11], in which he recorded a larva of *Gurelca montana* illustrated with a hand-drawn illustration. Unfortunately, after this illustration, the immature stages of this species have never been recorded again, and the pupal morphology and other detailed information, such as habits, remain unknown.

**Figure 1:**
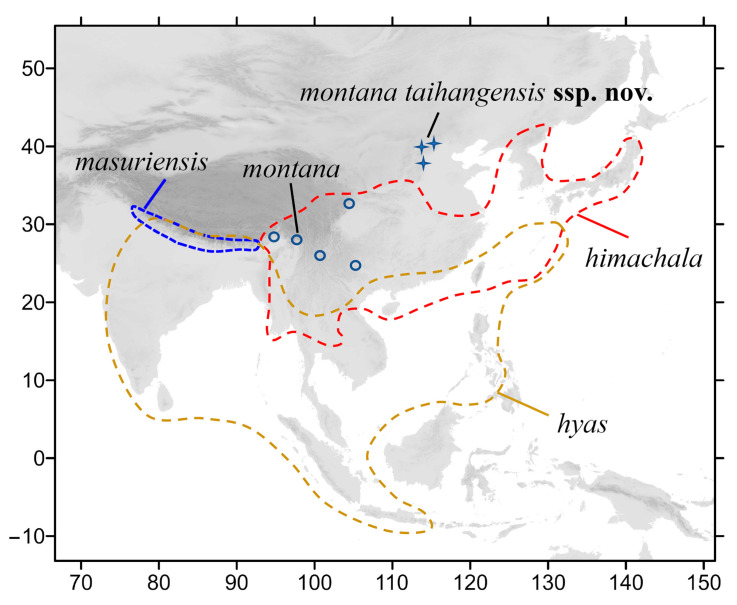
Distribution of species of genus *Neogurelca* in Asia. Ranges of three species are represented by dashed lines. The blue circles indicate the population of *N. montana* in Yunnan and Sichuan, the blue stars indicate the population of *N. montana* in Beijing and Hebei.

The crisp-banded hawkmoth, *Neogurelca himachala*, is closest in distribution to *N. montana* and is currently known to be distributed in the Himalayas; southwestern, central, and eastern China; the Korean peninsula; Japan; and northern Thailand [7,12,13]. This species has also been recorded in Beijing and Hebei [14,15,16] (Figure 1). However, these records must be treated as uncertain as they have not been confirmed by morphological and molecular studies.

Between 2018 and 2022, we collected Chinese *Neogurelca* specimens, especially *N. montana* and *N. himachala*, from different localities to clarify the differences between the species by morphological and phylogenetic analysis. After comparing the differences in wing patterns and genitalia, we found that the *Neogurelca* specimens from Beijing were not *N. himachala* as first thought but *N. montana*. Moreover, these features are also different between the *montana* population in Beijing and those in Yunnan and Tibet. We also obtained several larvae of *N. montana* from Beijing and recorded the larval food plant species and part of their life history for comparison with those of *N. himachala*. Our results support the conclusion that the population of *N. montana* from Beijing is relatively well-differentiated from those in Yunnan and Tibet, and we therefore describe and name it below as a new subspecies, with a review of all Chinese *Neogurelca* species.

## 2. Materials and Methods

### 2.1. Taxon Sampling

Specimens of the two focal species, *N. montana* and *N. himachala*, were sampled for both morphological and molecular analysis. Two specimens of *N. hyas* were also included to understand better the differences among the congeneric taxa in China. Most specimens were collected and dried in paper triangles and stored at −20 °C until use. Some samples were directly spread after collection to avoid abrasion of the scales of the head, thorax, and abdomen.

For each individual used in the molecular analysis, two legs from the same side were taken for DNA extraction before the specimens were rehydrated for spreading. Two sequences of the genus *Sphingonaepiopsis* Wallengren, 1858 were downloaded from the Barcode of Life Database v.4 (BOLD) (http://www.boldsystems.org (accessed on 11 October 2023)) as outgroups for phylogenetic analysis following the ‘*Sphingonaepiopsis* genus-group’, a putative monophyletic group identified by Kawahara et al. [17]. In addition, three sequences, including one individual of *N. himachala* and two individuals of *N. hyas,* were also downloaded from BOLD for this study. The collecting data, BOLD sample IDs, and GenBank accession numbers are listed in Table 1.

Due to difficulties obtaining fresh samples of the Indian and Nepalese species, *N. masuriensis*, this taxon was not included in the molecular analysis of this study but is discussed based on morphological characters derived from the original descriptions and photographs of type specimens (Appendix A).

### 2.2. DNA Extraction and Amplification

The phenol-chloroform protocol was used to extract genomic DNA. The legs were homogenized in protease buffer containing 450 μL STE (10 mmol/L Tris-HCl, 1 mmol/L EDTA, 100 mmol/L NaCl, pH = 8.0), 25 μL Proteinase K (20 mg/mL), and 75 μL SDS (10%) and incubated at 55 °C for 12 h to rehydrate and lyse the tissue. The subsequent extraction protocol followed Xu et al. [18] and the resultant genomic DNA was preserved at −40 °C.

DNA amplification followed Xu et al. [18]. The polymerase chain reaction (PCR) was carried out in a 25 μL system using the TaKaRa Ex *Taq* Kit (TaKaRa Biotechnology Co., Ltd., Dalian, China). The system contained 2.5 μL 10× PCR buffer, 2.0 μL MgCl_2_ (2.5 mmol/L), and 2.0 μL dNTP mixture (2.5 mmol/L each). The mitochondrial *cox1* gene fragment (the DNA barcode) was amplified and sequenced with the primers LCO1490 (5′-GGT CAA ATC ATA AAG ATA TTG-3′) and HCO2198 (5′-TAA ACT TCA GGG TGA CCA AAA AAT CA-3′) [19]. The PCR thermal profile consisted of an initial denaturation at 95 °C for 3 min, 30 cycles of denaturation at 94 °C for 1 min, annealing at 50 °C for 1 min, and elongation at 72 °C for 1 min; then a final elongation at 72 °C for 5 min. Sequencing was undertaken using an ABI Prism 3730 sequencer (Applied Biosystems, Foster City, CA, USA).

### 2.3. Phylogenetic Analysis and Species Delimitation

We proofread and aligned the raw sequences with Clustal W [20] in BioEdit 7.0.9 [21] by examining the chromatograms for polymorphic sites. MEGABLAST was used to check the identities of all sequences against genomic references and nucleotide collections in the BOLD and GenBank databases, and amino acid translation was performed with the invertebrate mitochondrial criterion in MEGA 7.0 [22] to detect possible *Numts* (nuclear copies of mtDNA fragments) [23,24]. A search for non-synonymous mutations, in-frame stop codons and indels was also carried out to detect possible cryptic *Numts*. The Kimura two-parameter distances [25] between taxa were calculated in MEGA 11 [26].

All sequences were included in the phylogenetic reconstructions without pruning identical haplotypes to test the phylogenetic integrity of the species as identified using morphological characters [27,28,29]. The phylogeny was reconstructed using a Bayesian Inference (BI) criterion as implemented in PhyloSuite 1.2.2 [30]. ModelFinder [31] was used to select the best fit model. We then used a Markov Chain Monte Carlo (MCMC) method to allow sampling across the entire substitution rate model space [32]. BI analysis consisted of two independent runs, each with eight MCMC running for five million generations (sampled every 1000th generation) to calculate the clade posterior probabilities (PP). The marginal likelihood estimate was performed with stepping-stone sampling [33], as implemented in MrBayes with 100 steps, each with 10 million generations, and a diagnostic frequency of 1000. Finally, Monophylizer (http://monophylizer.naturalis.nl/ (accessed on 11 October 2023)) was applied to the resultant Bayesian tree to test the monophyly of each identified taxon [34].

### 2.4. Morphological Comparison

Specimens were spread for morphological comparison before molecular analysis based on habitus. Each specimen was photographed using a Canon EOS 90D digital camera (Canon, Japan), and the exposure was adjusted using Adobe Photoshop CS (Adobe, San Jose, CA, USA).

The male and female genitalia were prepared following Hu et al. [35]. Forewing lengths were measured to 0.5 mm precision using a ruler. The whole abdomen was removed and placed into a 1.5 mL microcentrifuge tube, treated with 1 mL 10% sodium hydroxide solution to digest soft tissue for 1 h at 70 °C. The treated abdomen was then neutralized with 2% acetic acid and dissected in a water-filled Petri dish under a stereomicroscope to remove residual tissues, scales, and hair. The genitalia were transferred to 80% glycerol for 12 h to render them transparent. Photographs of the genital structures were taken with a KUY NICE E3ISPM (KUY NICE, Beijing, China) and automatically stacked using Helicon Focus 7.5.4 (Helicon Software, Kharkiv, Ukraine). After examination and photographing, all parts of the genitalia were fixed onto a card with water-soluble white glue and pinned with the specimen.

### 2.5. Larvae Collection and Rearing

Three final instar larvae were collected, one each from the lower mountain area of Huangshandian village, the Beijing Shangfangshan National Forest Park (Fangshan District, Beijing, China), and the China National Botanical Garden after observing larvae on the food plants (*Leptodermis oblonga* Bunge) in the field. Tender shoots bearing larvae were clipped from the food plants and placed individually into plastic boxes lined with moist tissue on site. Branches of the food plant with healthy leaves were also collected and sealed in plastic bags and preserved at 4 °C for future use.

The rearing experiment was performed in downtown Beijing without use of a climate chamber. Larval frass and leaf residue was removed from the boxes and the moist tissue was changed each day to keep the environment clean. The body length of each larva was measured to a 1 mm precision. A lateral view photograph was taken of the larva and pupa, and an additional dorsal view photograph was also taken for the pupal chamber.

Only one larva developed to an adult. Due to the limited number of samples available for this study, no statistical analysis was made of body length and duration of each instar, and no acceptance tests were made with other possible food plants.

## 3. Results

### 3.1. Molecular Phylogenetic Relationships and Delimitation

#### 3.1.1. Molecular Phylogenetic Analysis

**Figure 2:**
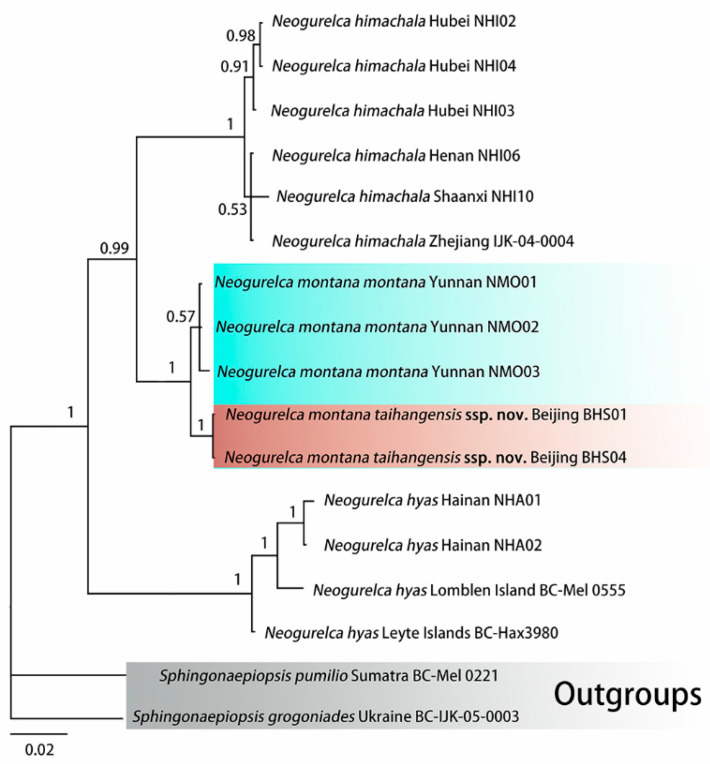
Bayesian Inference phylogenetic tree of *Neogurelca* based on the DNA barcode sequences (*cox1*) and rooted on *Sphingonaepiopsis gorgoniades* and *S. pumilio* as outgroups. Values at the nodes indicate posterior probabilities.

ModelFinder selected the best fit model as GTR+F+G4. Bayesian phylogenetic analysis converged well, as indicated by an average standard deviation of split frequencies close to 0 (0.002874), potential scale reduction factors equal to 1 (maximum = 1.003), and an effective sample size (ESS) >> 200 for all parameters. The phylogeny recovered the three species of *Neogurelca* as monophyla, each with a maximal PP value (Figure 2).

The first clade comprises *N. himachala* collected from different localities. The second clade comprises the reciprocally monophyletic *N. montana montana* and *N. montana taihangensis* **ssp. nov.** and *N. himachala* and *N. montana* are sister species. Sister to these two taxa is a clade comprising *N. hyas* from Lomblen Island (Indonesia), Leyte Island (Philippines), and Hainan Island (China) (Figure 2).

#### 3.1.2. Molecular Species Delimitation

The Kimura two-parameter (K2P) distances among the taxa ranged from 0.9 to 10.0%, with that between *N. montana montana* and *N. montana taihangensis* **ssp. nov**. being the smallest (0.9%), and that between *N. himachala* and *N. hyas* being the largest (10.0%) (Table 2). The distance between the new subspecies and *N. montana montana* is much lower than those between the other known *Neogurelca* species.

The Monophylizer analysis supported all morphologically recognized species and subspecies as monophyletic on the BI tree (Table 3). The results showed that all samples of the new subspecies also formed a monophyletic group.

Given this molecular evidence, we propose that the *Neogurelca montana* from Beijing is a new subspecies and describe it below.

### 3.2. Morphological Comparison

**Abbreviation for specimen storage institution**

Names of institutions in which specimens are deposited are listed after private collections and are abbreviated as follows: CQL—collection of Chang-Qiu Liu (Guilin, China); ZBX—collection of Zhen-Bang Xu (Guilin, China); TL—collection of Tao Li (Beijing, China); ZHJ—collection of Zhuo-Heng Jiang; NHMUK—collections of the Natural History Museum (London, United Kingdom); KIZ—collections of the Kunming Institute of Zoology, Chinese Academy of Science (Kunming, Yunnan, China); IOZ—collections of the National Animal Collection Resource Center, Institute of Zoology, Chinese Academy of Sciences.

#### 3.2.1. *Neogurelca montana montana* (Rothschild & Jordan, 1915)

Material examined: **SYNTYPES:** 4♂♂, Thibet [Tibet, China] (without more definite locality and time information), Le Moult *leg*. [NHMUK 014200047, NHMUK 014200048, NHMUK 014200049 and NHMUK 014200050];

**Additional specimens:** ♂, Shangri-La Alpine Botanical Garden (3300 m), Diqing, Yunnan, China, 2017-VIII, Chang-Qiu Liu *leg*. [ZBX]; ♂, Bingzhongluo, Nujiang, Yunnan, China, 2021-VII-26 Si-Yao Huang *leg*. [KIZ 0132781]; ♀, Cang Shan (2200 m), Dali, Yunnan, China, 2020-XI-9, Lan-Fang Zhao *leg*. [ZBX]; ♀, Nyingchi (2400 m), Tibet, China, 2021-VI-4, Guo-Cong Huang *leg*. [ZHJ]; ♀, Cang Shan (2350 m), Dali, Yunnan, China, 2021-X-14, Zhang Wei *leg*. [ZHJ].

**Description:**

**Figure 3:**
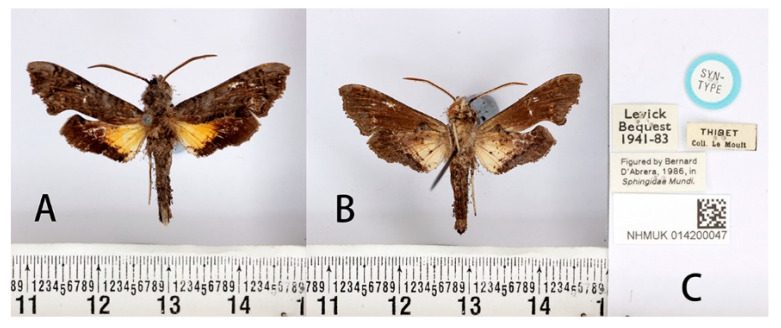
Syntype of *Neogurelca montana montana* (Rothschild & Jordan, 1915), Tibet, China, male. Photo by A. Giusti, NHMUK. (**A**) Upperside; (**B**) underside; (**C**) labels. All images © The Trustees of the Natural History Museum, London, UK.

**Figure 4:**
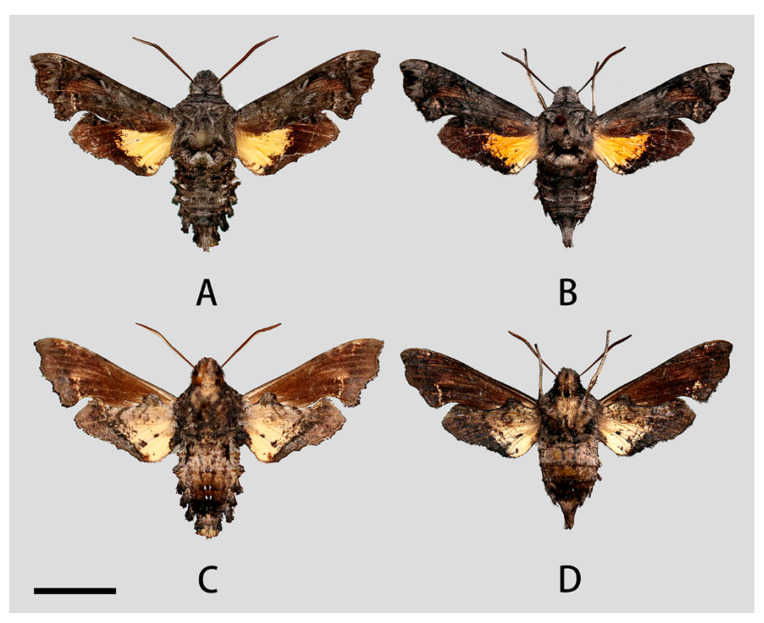
Photos of *Neogurelca montana montana*. (**A**,**C**) Male, Shangri-La Alpine Botanical Garden, Diqing, Yunnan, China; (**B**,**D**) female, Cang Shan, Dali, Yunnan, China. Scale bar = 10 mm.

**Figure 5:**
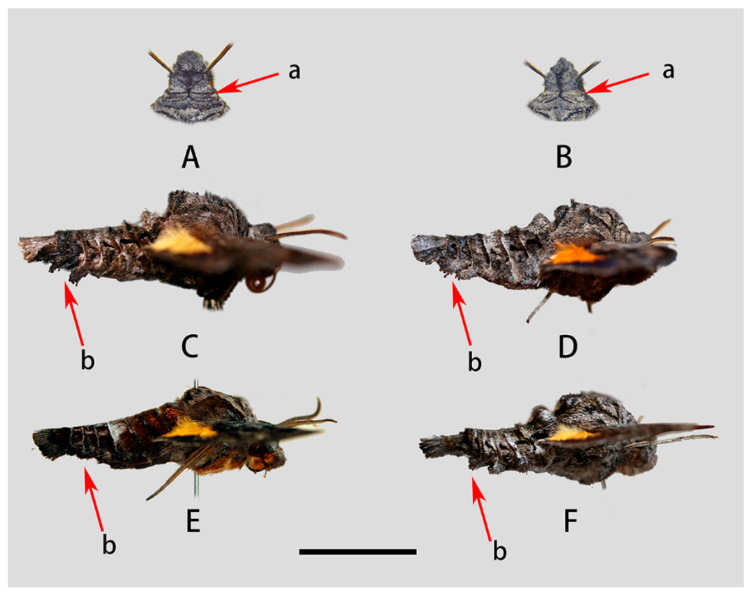
Morphological comparison of *Neogurelca montana montana*, *N. montana taihangensis* Xu & He **ssp. Nov.** and *N. himachala*. (**A**) Head of *N. montana montana*; (**B**) head of *N. montana taihangensis* Xu and He **ssp. Nov.**; (**C**) lateral view of male *N. montana montana*; (**D**) lateral view of male *N. montana taihangensis* Xu and He **ssp. Nov.**; (**E**) lateral view of male *N. himachala*; (**F**) lateral view of female *N. montana taihangensis* Xu and He **ssp. nov.**; a—patagia; b—lateral hair tufts in abdomen. Scale bar = 10 mm.

**Male** (Figure 3 and Figure 4A,C). Forewing length 19 mm, silky ashen-grey, without reddish-brown markings on the thorax seen in other congeneric species. Body ashen-grey. Antenna about half of forewing length. Patagia with pair of flabellate patches on each side, conspicuously bordered in black (Figure 5A). Abdominal tergites 5, 6, and 7 with lateral hair tufts on each side (Figure 5C). Abdomen ending in a fan-shaped tuft. Forewing upperside: Ground colour ashen-grey, with a dark oblique band from costa towards tornal angle, bordered on the outer side with white, reaching vein M_3_. Discal white band short and oblique. Terminal band inwardly wavy with black boundary. Forewing underside: dark brown as far as the postdiscal line at inner angle, the postmedial line thin, brownish-yellow. Hindwing upperside: Blackish-brown, yellow fan-like patch from base to half of hindwing, with fuzzy outer boundary. Hindwing underside: Ashy greyish-brown, anal area pale straw-coloured.

**Female** (Figure 4B,D). Forewing length 19 mm, external morphology similar to male but forewing with blunter apex. Abdomen ending thinner than male.

**Figure 6:**
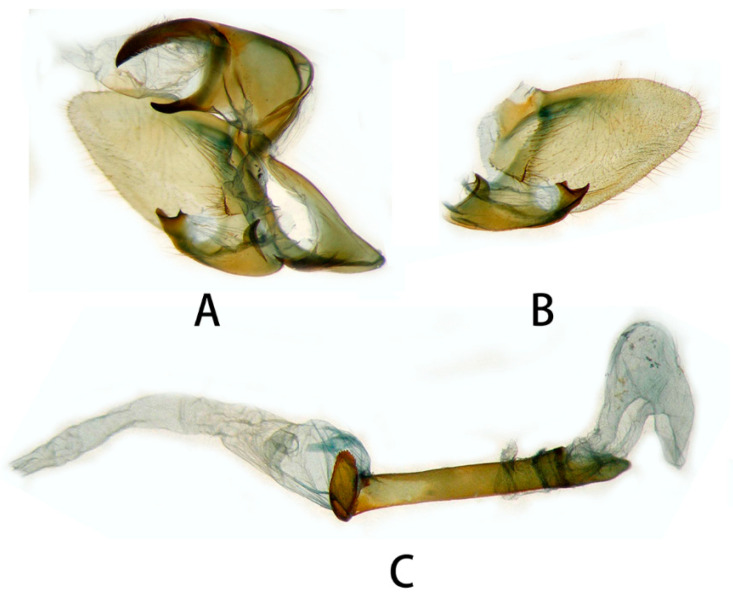
Male genitalia of *Neogurelca montana montana*, Yunnan, China. (**A**) Lateral view; (**B**) right valve; (**C**) phallus. © The Trustees of the Natural History Museum, London, UK. https://sphingidae.myspecies.info/taxonomy/term/1965/media (accessed on 11 October 2023).

**Figure 7:**
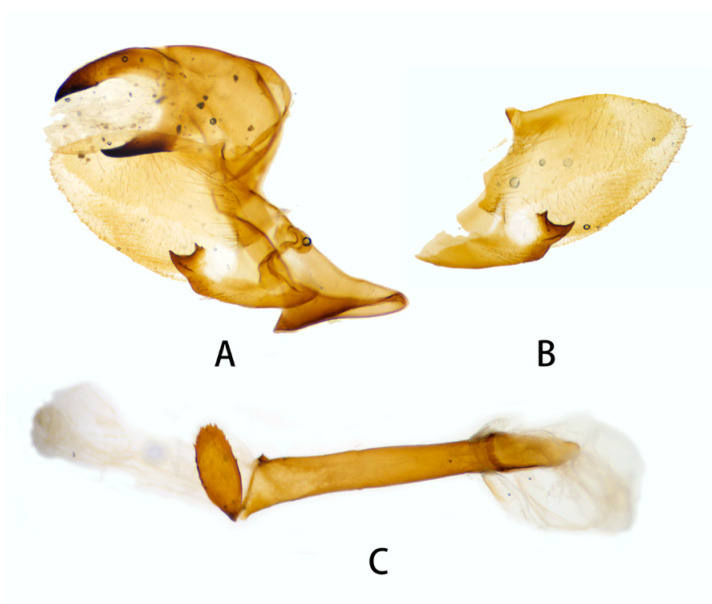
Male genitalia of *Neogurelca montana montana*, Nujiang, Yunnan, China. (**A**) Lateral view; (**B**) right valve; (**C**) phallus.

**Male genitalia** (Figure 6 and Figure 7). Uncus and gnathos forming a typical macroglossine ‘bird-beak’ structure. Uncus slightly curved, with a tiny apical hook. Gnathos short, curved as uncus, apex slightly upward. Valve leaf-shaped with broader basal part, gradually narrowed to the blunt apex. Sacculus curved, extended apically into a highly sclerotized harpe with two sharp processes. Phallus long and slender, with a distal transverse process; the transverse apical process oval-shaped with tiny upwardly directed spinules.

**Figure 8:**
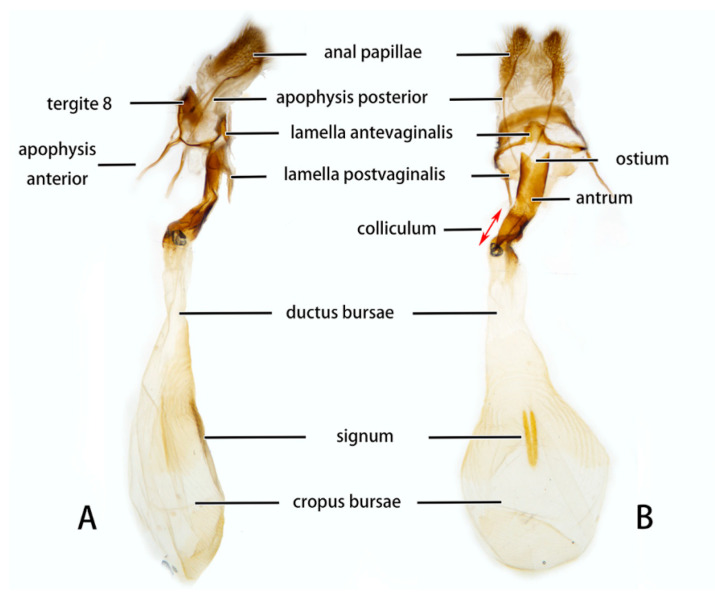
Nomenclature of female genitalia of *Neogurelca montana montana*, Dali, Yunnan, China. (**A**) Lateral view; (**B**) ventral view.

**Female genitalia** (Figure 8). Anal papillae short, consisting of two ridge-shaped lobes with long setae; posterior apophysis slender, arising from the tergite 10 laterally and reaching ostium bursae; lamella antevaginalis ridge-shaped medially, with the anterior apophyses arising on each side; lamella postvaginalis composed of two slightly sclerotized and separated lobes; ostium bursae heavily sclerotized, inverted triangle-shaped; antrum long and broad, extended by a quite heavily sclerotized and twisted colliculum; ductus bursae long and gradually broadening into a rounded corpus bursae; signum well defined by sclerotized granules.

**Voltinism**: Uncertain, adults appear from late July to November.

**Distribution**: Currently known from northeast Sichuan, eastern and northwestern Yunnan, and southeast Tibet.

**Biology**: Adults are fairly common between July and October on grassy slopes at 2000–2400 m elevation [11]. They are only active at dusk and only occasionally attracted by light. At rest, adults fold the costal margin of hindwing up over the leading edge of the forewing. Overall, it then looks like a dead branch. In Yunnan, the larval host plant is *Paederia foetida* (Rubiaceae) [11,36].

#### 3.2.2. *Neogurelca montana taihangensis* Xu & He ssp. nov.

LSID urn:zoobank.org:act:A021EEC4-B226-41BA-8536-782BD04E5BD0

**HOLOTYPE**: ♂, Huangshandian village (130 m), Fangshan District, Beijing, China, 2022-IX-13, Zhen-Bang Xu *leg*. [KIZ 0132780]. **PARATYPES**: ♂, Beijing Baihuashan National Nature Reserve (1400 m), Mentougou District, Beijing, China, 2018-X, Y. Nan *leg*. [ZBX]; ♂, Xiaolongmen Forest Farm (1100 m), Mentougou District, Beijing, China, 2021-VIII, Hong-Lin Zhu *leg*. [ZBX]; ♂, same locality, 2022-VI-5, Hong-Lin Zhu *leg*. [ZBX]; ♂, China National Botanical Garden, Haidian District, Beijing, China, 2022-X-1, Yu-Chen Zhang *leg*. [ZBX]; ♂, Yue’an mountain forestry centre of Beijing University of Agriculture (800 m), Sidaohe Village, Baoshan Town, Huairou District, Beijing, China, 2022-VIII-15, Tao Li *leg*. [IOZ 221490] [37]; ♀, Beijing Baihuashan National Nature Reserve (1400 m), Mentougou District, Beijing, China, 2013-VI, Nan Yang *leg*. [ZBX]; ♀, Yunmengshan National Forest Park, Minyun District, Beijing, China, 2018-VII, Chen Wang *leg*. [ZBX]; ♀, China National Botanical Garden, Haidian District, Beijing, China, 2022-X-1, Yu-Chen Zhang *leg*. [ZBX].

**Description and Diagnosis:**

**Figure 9:**
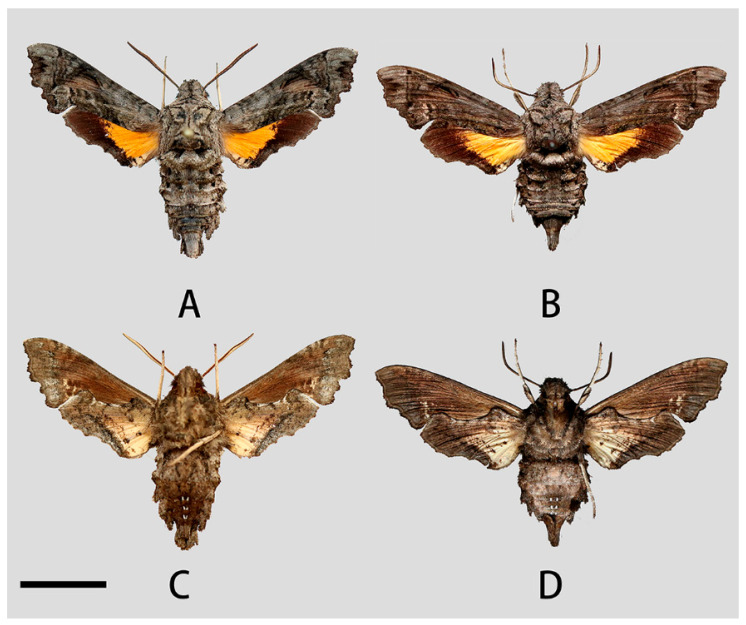
Photos of *Neogurelca montana taihangensis* Xu & He **ssp. nov.** (**A**,**C**) ♂, HOLOTYPE, Fangshan, Beijing, China; (**B**,**D**) ♀, PARATYPE, Mentougou, Beijing, China. Scale bar = 10 mm.

**Male** (Figure 9A,C). Forewing length 17–20 mm, similar to the nominotypical subspecies but can be distinguished by the following characters: (1) in *N. montana taihangensis* **ssp. nov.**, body and forewing paler than *N. montana montana*; (2) the yellow patches of the hindwing of the new subspecies are triangular shaped, with more distinct outer boundary; and (3) the anterior boundary of patagium in *taihangensis* **ssp. nov.** is fuzzier than that of the nominotypical subspecies (Figure 5A,B).

**Female** (Figure 9B,D). Forewing length 18–19 mm, external morphology similar to male. Abdomen apex much thinner than the male.

**Figure 10:**
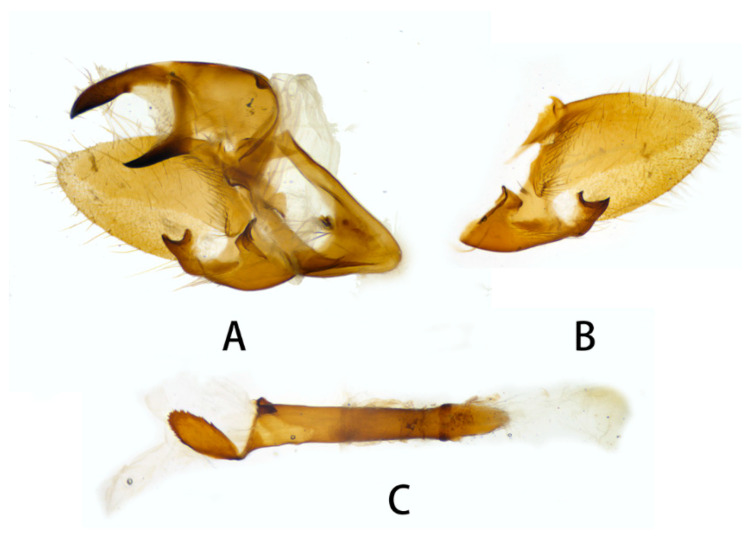
Male genitalia of *Neogurelca montana taihangensis* Xu & He **ssp. nov.**, Fangshan, Beijing, China. (**A**) Lateral view; (**B**) right valve; (**C**) phallus.

**Male genitalia** (Figure 10). Same as *N. montana montana*, but the width of the transverse apical process of phallus in the new subspecies is narrower than that of the nominotypical subspecies.

**Figure 11:**
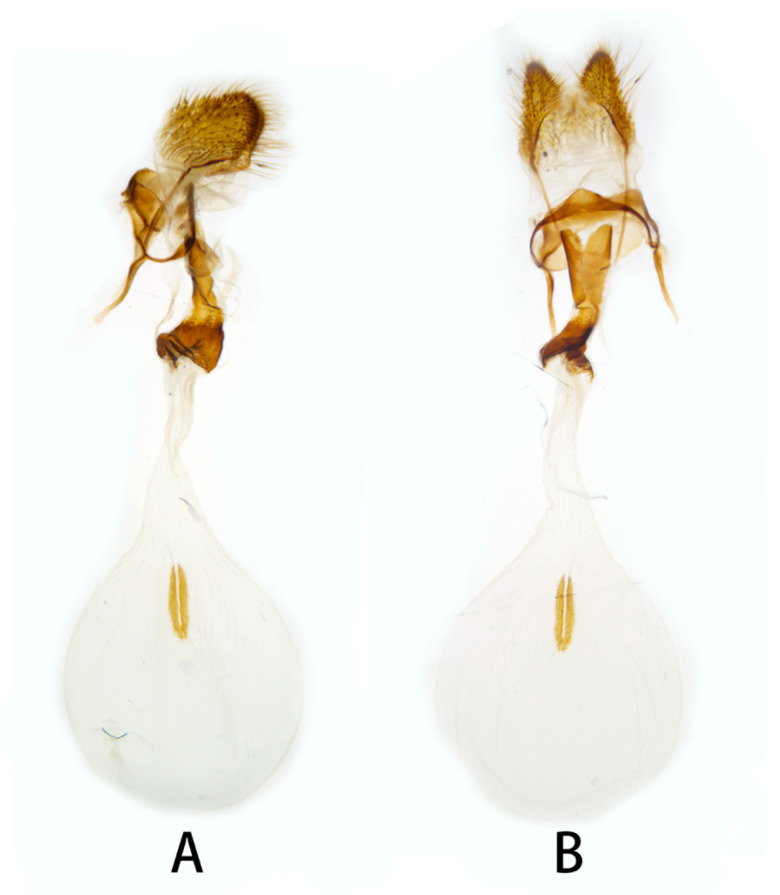
Female genitalia of *Neogurelca montana taihangensis* Xu & He **ssp. nov.**, Haidian, Beijing, China. (**A**) Lateral view; (**B**) ventral view.

**Female genitalia** (Figure 11). Same as *N. montana montana*.

**Voltinism**: Bivoltine, the first generation appearing from March to July, the second generation appearing from August to November.

**Distribution**: Currently known in Beijing, central and southern Hebei, coinciding with the Taihang Mountains.

**Derivatio nominis**: The subspecific epithet derives from the Taihang Mountains, as all specimens have been collected in this area (the name is to be treated as a noun in apposition).

**Biology**: Unlike the population of Yunnan and Tibet, adults rarely appear in numbers. In Beijing, adults fly in low mountainous areas and urban parks at 100 m elevation, but also in mountains at 1500 m elevation. Larvae feed on *Leptodermis oblonga* Bunge around Beijing.

#### 3.2.3. *Neogurelca himachala* (Butler, 1876)

**Description:**

**Figure 12:**
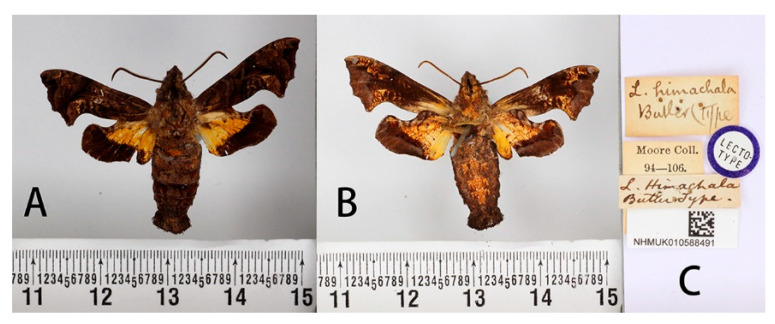
Lectotype of *Neogurelca himachala* (Butler, 1876), India, male. Photo by A. Giusti, NHMUK. (**A**) Upperside; (**B**) underside; (**C**) labels. All images © The Trustees of the Natural History Museum, London, UK.

**Figure 13:**
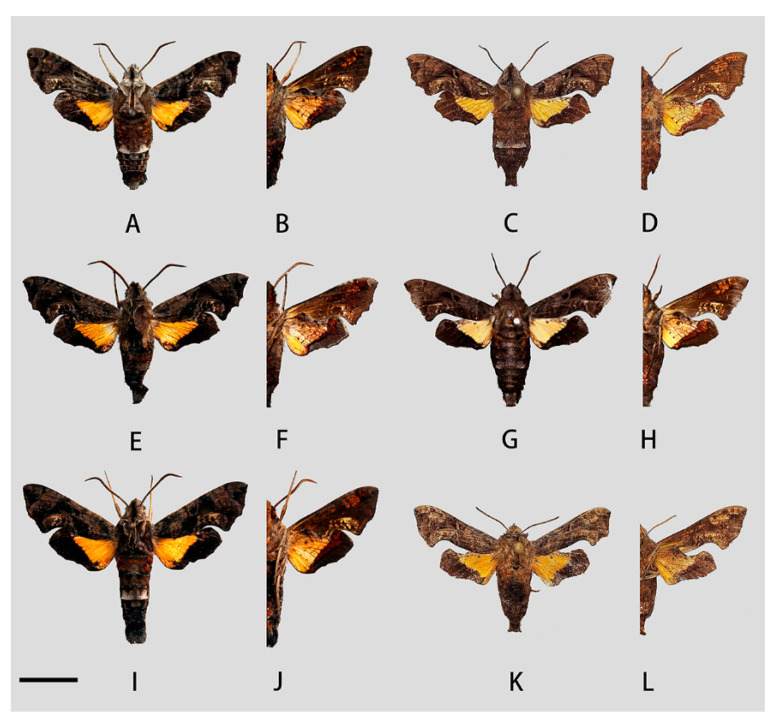
Photos of *Neogurelca himachala* from different localities. (**A**,**B**) Male, Wuhan Hubei, China; (**C**,**D**) female, Hangzhou, Zhejiang, China, © Zhuo-Heng Jiang; (**E**,**F**) male, Henan, China; (**G**,**H**) ♂, Shaanxi, China, © Chang-Qiu Liu; (**I**,**J**) male, Guilin, Guangxi, China; (**K**,**L**) female, Yuanjiang, Yunnan, China, © Zhuo-Heng Jiang. Scale bar = 10 mm.

**Male** (Figure 12 and Figure 13A,B,E–J). Forewing length 16–19 mm, dark brown. Head and body dark brown, some individuals greyish-brown on the head and thorax. A black vertical line running medially from head to thorax. Dark brown triangular patches with grey margin on each side of thorax. On the abdomen, tergite 1 and 2 with orange tufts in each side, and a white ridge-shaped patch on each side of tergite 4 in some individuals. Antenna about half as long as the forewing. Abdomen ending in a brush-shaped tuft. Forewing upperside: Brownish, with scattered black patches. Postmedial band black, slender and curved. Discal spots black with hazel oblique short line. A light brown oval patch on the tornal area, with scattered golden spots inside. Forewing underside: dark brown, with orange scattered spots gathered at costal margin and inner margin. Hindwing upperside: dark brown, a well-defined triangular yellow patch from base to half of hindwing. Hindwing underside: dark brown, yellow patch with red margin.

**Female** (Figure 13C,D,K,L). External morphology same as male.

**Figure 14:**
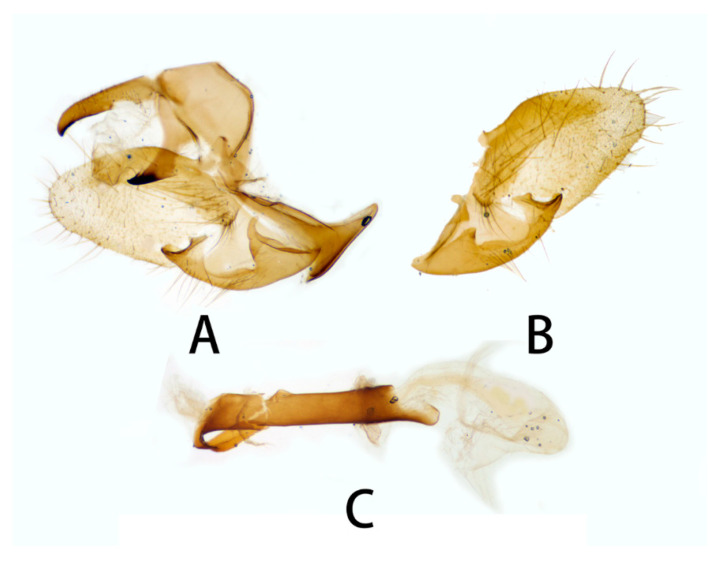
Male genitalia of *Neogurelca himachala*, Nanjing, Jiangsu, China. (**A**) Lateral view; (**B**) right valve; (**C**) phallus.

**Male genitalia** (Figure 14). Uncus and gnathos forming a ‘bird-beak’ structure. Uncus slenderer than *N. montana*, with a slightly curved apical hook, upper edge flat. Gnathos shorter than *N. montana*, apex slightly blunt. Valve leaf-shaped, with narrower basal part, gradually broader to half of valve, then narrower to apex, apex blunt. Sacculus with a hollow basal process, open above, the harpe distally raised into a sinuate ridge. Phallus long and slender, with a long flat process, curving proximad round the sheath and lying flat on it, the proximal edge of this process with vestigial denticulations.

**Figure 15:**
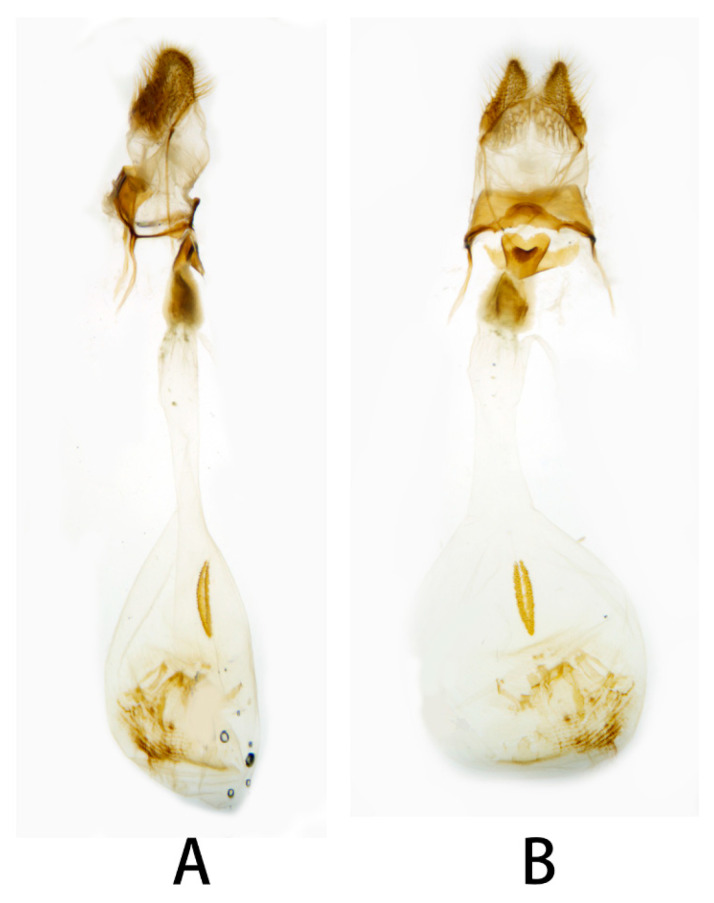
Female genitalia of *Neogurelca himachala*, Shaanxi, China. (**A**) Lateral view; (**B**) ventral view.

**Female genitalia** (Figure 15). Anal papillae short, consisting of two ridge-shaped lobes with long setae; posterior apophysis slender, arising from the tergite 10 laterally and reaching ostium bursae; lamella antevaginalis ridge-shaped with broader and blunt posterior edge, lined with the anterior apophyses arising on each side; lamella postvaginalis composed of two slightly sclerotized and separated lobes; ostium bursae heavily sclerotized, heart-shaped; antrum short with a slightly sclerotized, followed by a short colliculum, which has two sclerotized tubes inside; ductus bursae long and gradually broadening into a rounded corpus bursae; signum well defined by sclerotized granules.

**Voltinism**: Unclear, adults can be found from March to October.

**Distribution**: Central, eastern and southwestern China, North Korea, South Korea, Japan, Nepal, northeastern India, and northern Thailand.

**Biology**: The adults do not fly very much and are only active for a few hours just after daybreak and at dusk. Occasionally attracted by light.

A noisy flier that can manoeuver with precision in and out of branches and undergrowth, but which will dart off at high speed if disturbed. Adults hide in leaves or bark during the day while resting. The patterns and colours of the wings blend well with the background. Larvae feed on *Paederia foetida* (Rubiaceae) in China.

#### 3.2.4. *Neogurelca hyas* (Walker, 1856)

**Description:**

**Figure 16:**
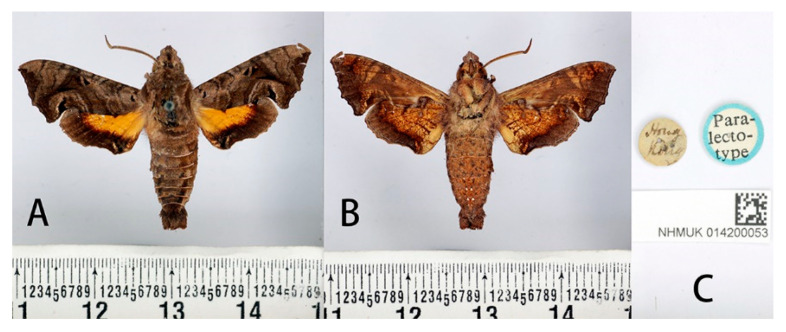
Paralectotype of *Neogurelca hyas* (Walker, 1856), Hong Kong, China, male. Photo by A. Giusti, NHMUK. (**A**) Upperside; (**B**) underside; (**C**) labels. All images © The Trustees of the Natural History Museum, London, UK.

**Male** (Figure 16). Forewing length 18 mm, head and body greyish-brown. Head with a black and thick vertical line medially. Dark brown triangular patches with greyish-brown margin on each side of thorax. Abdomen with reddish-brown, segmental markings on both sides. Antenna about half the forewing length. Abdomen ending in a brush-shaped tuft. Forewing upperside: Greyish-brown, with a black spot basally and two pale, indistinct, curved, antemedial lines. Discal spots black with hazel oblique short line. Two highly angulate postmedial lines with a pale line between them from CuA_1_ to inner margin, and a reddish-brown streak below M_3_. A light brown oval patch on tornal area, with a black curved line inside. Forewing underside: ochreous, with orange scattered spots gathered at costal margin and inner margin. Hindwing upperside: Brownish, with yellow oval patch basally, brownish band of even width along outer margin. An annular spot on the discocellular veins. Hindwing underside: ochreous, much marbled and suffused with brown and reddish-brown.

**Figure 17:**
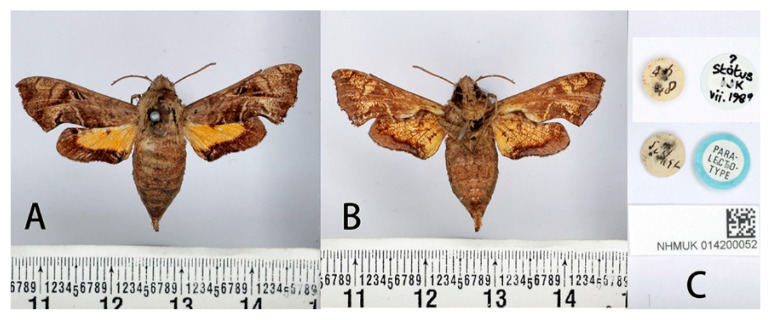
Paralectotype of *Neogurelca hyas* (Walker, 1856), Sylhet, Bangladesh, female. Photo by A. Giusti, NHMUK. (**A**) Upperside; (**B**) underside; (**C**) labels. All images © The Trustees of the Natural History Museum, London, UK.

**Female** (Figure 17). External morphology same as male, but abdomen apically much thinner.

**Figure 18:**
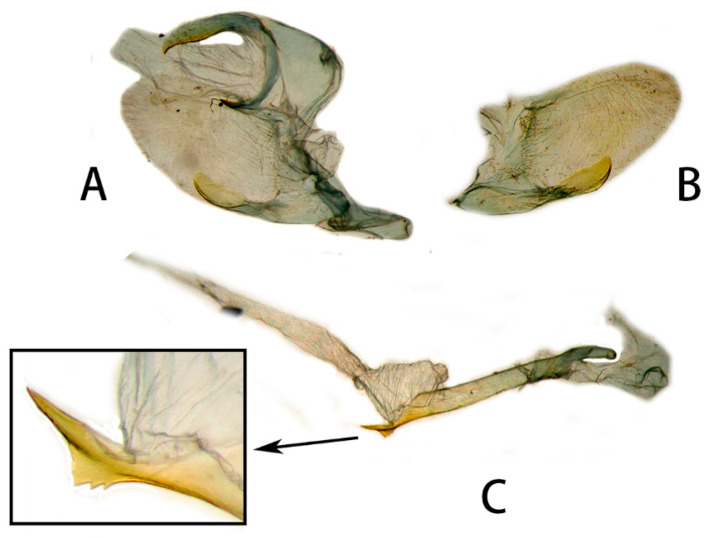
Male genitalia of *Neogurelca hyas*, Hainan, China. (**A**) Lateral view; (**B**) right valve; (**C**) phallus. © The Trustees of the Natural History Museum, London, UK. https://sphingidae.myspecies.info/taxonomy/term/1961/media (accessed on 11 October 2023).

**Male genitalia** (Figure 18). Uncus and gnathos forming a ‘bird-beak’ structure. Uncus slender, with a slightly curved apical hook, swollen medially. Gnathos short and pointed. Valve oval. Harpe paddle-shaped, with a narrow base. Phallus long and slender, with a distal transverse process; the transverse apical process curved with distinct dentation along one side.

**Figure 19:**
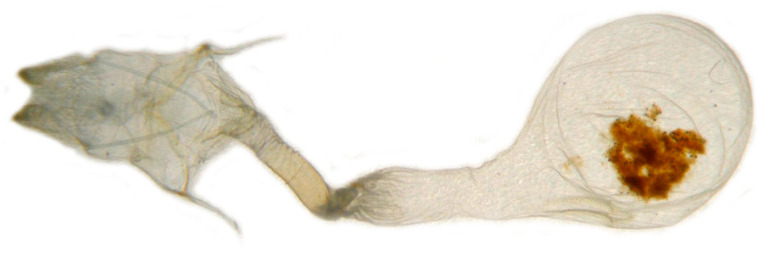
Female genitalia of *Neogurelca hyas*, Assam, India. © The Trustees of the Natural History Museum, London, UK. https://sphingidae.myspecies.info/taxonomy/term/1961/media (accessed on 11 October 2023).

**Female genitalia** (Figure 19). Anal papillae short, consisting of two ridge-shaped lobes with long setae; posterior apophysis slender, arising from the tergite 10 laterally and reaching ostium bursae; lamella antevaginalis slightly sclerotized, inverted ‘V’ shaped, with anterior apophyses arising on each side; lamella postvaginalis composed of two slightly sclerotized and separated lobes; ostium bursae sclerotized, indented; antrum short and plicate, lined leading to a slender colliculum; ductus bursae long and gradually broadening to a rounded corpus bursae; signum well defined by sclerotized granules.

**Voltinism**: Unclear, adults can be found from February to November.

**Distribution**: India, Nepal, Bhutan, Myanmar, southern China, southern Japan, Thailand, Laos, Vietnam, Malaysia, Indonesia, and Philippines.

**Biology**: Adults fly at dusk. Larvae can be found on *Paederia foetida* and *Serissa foetida* (Rubiaceae) in southern China.

#### 3.2.5. *Neogurelca masuriensis* (Butler, 1875)

**Description:**

**Figure 20:**
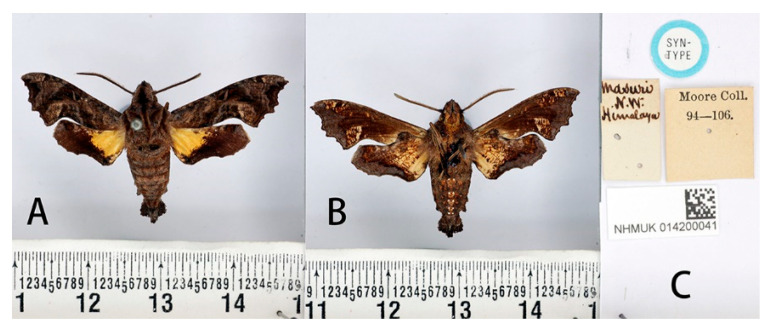
Syntype of *Neogurelca masuriensis* (Butler, 1875), India, male. Photo by A. Giusti, NHMUK. (**A**) Upperside; (**B**) underside; (**C**) labels. All images © The Trustees of the Natural History Museum, London, UK.

**Male** (Figure 20). Forewing length 19–20 mm, head and body greyer than *N. himachala*. A black vertical line running medially from head to thorax. Abdomen with hazel markings on both sides. Antenna about half of forewing length. Abdomen ending in a fan-shaped tuft. Forewing upperside and underside similar to *N. himachala*, but on the forewing upperside: anal lobe shorter than *himachala*, the oval patches on tornal area distinctly divided into two parts, the inner part taupe, and the outer part yellowish-brown. On the hindwing upperside: the black border narrowing behind, not sharply defined on the inner side, diffused onto the disc; the yellow patches fan-shaped.

**Figure 21:**
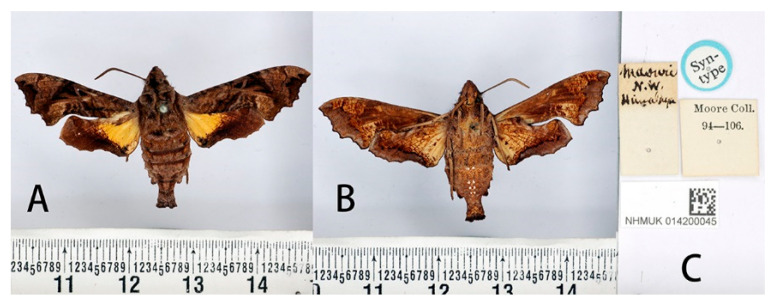
Syntype of *Neogurelca masuriensis* (Butler, 1875), India, female. Photo by A. Giusti, NHMUK. (**A**) Upperside; (**B**) underside; (**C**) labels. All images © The Trustees of the Natural History Museum, London, UK.

**Female** (Figure 21): External morphology similar to male, but abdomen apically much thinner.

**Figure 22:**
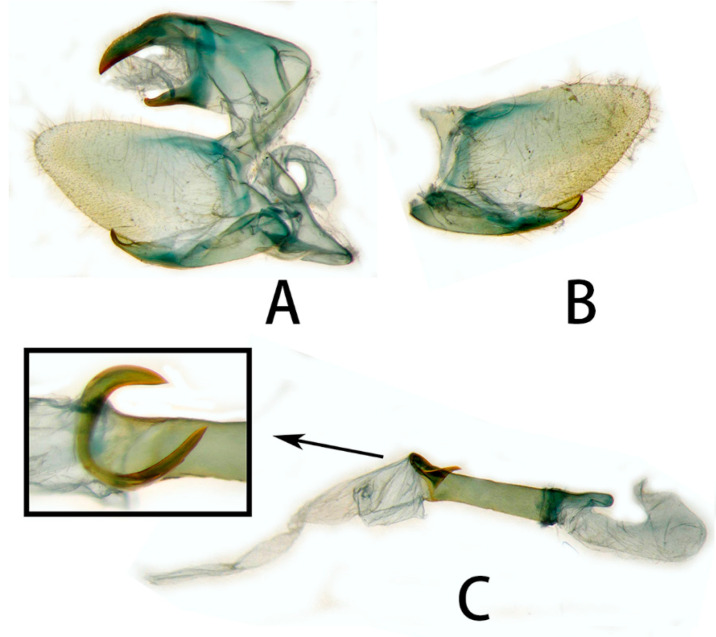
Male genitalia of *Neogurelca masuriensis*, India. (**A**) Lateral view; (**B**) right valve; (**C**) phallus. © The Trustees of the Natural History Museum, London, UK. https://sphingidae.myspecies.info/taxonomy/term/1963/media (accessed on 11 October 2023).

**Male genitalia** (Figure 22). Uncus and gnathos forming a ‘bird-beak’ structure. Uncus broad, heavily downward curved in base, gradually narrowed to a point. Gnathos broad at base, with a blunt upward apex. Valve ligule-shaped. Harpe spatulate, concave on upperside, apical margin incised or emarginate above middle. Phallus long and slender, with a distal transverse process, which consists of a prominent non-dentate ridge, ending on both sides in a sharp hook directed frontad, overall like a crescent.

**Figure 23:**
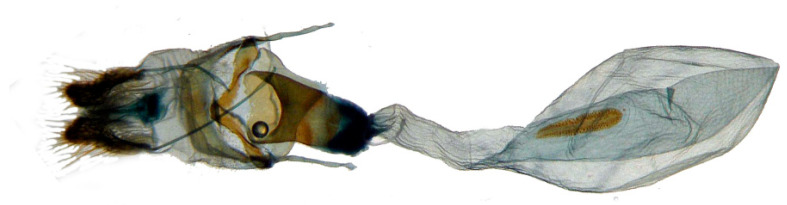
Female genitalia of *Neogurelca masuriensis*, India. © The Trustees of the Natural History Museum, London, UK. https://sphingidae.myspecies.info/taxonomy/term/1963/media (accessed on 11 October 2023).

**Female genitalia** (Figure 23). Anal papillae short, consisting of two ridge-shaped lobes with long setae; posterior apophysis slender, arising from the tergite 10 laterally and reaching ostium bursae; lamella antevaginalis sclerotized, inverted ‘V’ shaped, with anterior apophyses arising on each side; lamella postvaginalis composed of two slightly sclerotized and separated lobes; ostium bursae sclerotized, broad and indented; antrum short and broad, lined with slender colliculum; ductus bursae long and gradually broadened and merging into the rounded corpus bursae; signum well defined by sclerotized granules.

**Voltinism**: Unknown.

**Distribution**: From northwestern India along the southern Himalaya of northern India to Bhutan [Irungbam & Irungbam, 2019] [38].

**Biology**: Larval host plant is *Leptodermis lanceolata* (Rubiaceae) [36].

#### 3.2.6. Diagnostic Characters

In external appearance: *N. montana* can be easily distinguished by the absence of brown markings in thorax found in the other congeneric species. In male genitalia: *N. montana* are closest to *N. himachala*, but (1) the upper edge of uncus in *montana* curved than that of *himachala*. (2) Gnathos shorter in *himachala* than in *montana*. (3) The distal transverse process of the two in differently shaped. It is oval in *montana*, sheath-shaped in *himachala*; the male genitalia of *montana* are similar to those of *hyas*, but (1) gnathos shorter in *hyas* than in *montana*. (2) Harpe of *hyas* paddle-shaped, *montana* has two sharp upwardly directed points. (3) The distal transverse process is curved in *hyas*; that of *montana* is similar to *masuriensis*, but (1) harpe of latter spatulate. (2) The distal transverse process crescent-shaped in *masuriensis*.

In the female genitalia: *N. montana* can be easily distinguished by the long and twisted colliculum compared with that of other congeneric species.

### 3.3. Life Histories of N. montana taihangensis *ssp. nov.* and N. himachala

#### 3.3.1. *N. montana taihangensis* ssp. nov.

**Figure 24:**
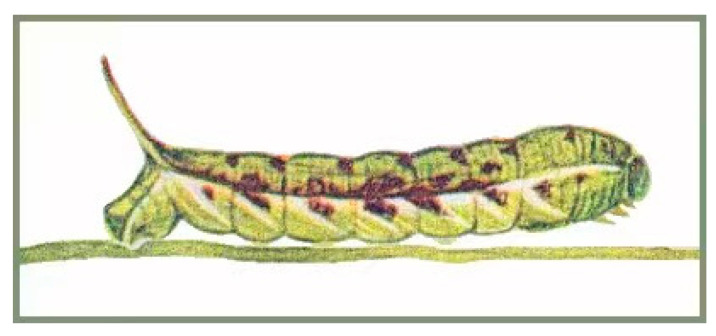
Larva of *Neogurelca montana montana*, Dali, Yunnan, China. Painting from Mell [11].

**Figure 25:**
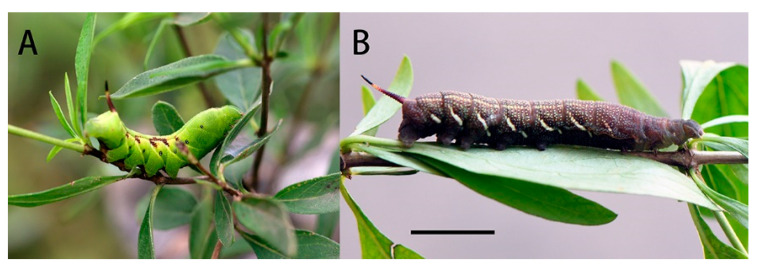
Larval photo of *Neogurelca montana taihangensis* Xu & He **ssp. nov.** (**A**): Final instar larva; (**B**) prepupa. Scale bar = 10 mm.

Final instar larva: Same as *N. montana montana* [36] (Figure 24). The final instar larva 41–43 mm, green with rust-brown markings. Head green, nearly oval, with a whitish stripe separating face from cheek. Surface dull and set with short, curved, translucent hairs, each rising from a minute tubercle (Figure 25).

Body green, dull, smooth, and cylindrical, thoracic segments flared laterally into flanges. True legs pale green, rusty-red medially. A yellow dorso-lateral stripe runs from segment 2 to base of horn and thence onto 13, enclosing a rust-brown dorsal patch behind the horn. There are pale oblique lateral stripes in abdominal segments 1 to 7, the angles formed by the junction of the oblique stripes with the subdorsal stripe filled in with rusty-red, and the subdorsal stripe edged above with rusty-red near these junctions. Spiracles black with two white, central slits in upper and lower positions.

Horn slate grey with a pale-yellow ring beyond the middle. Surface covered with very regularly placed, small black tubercles. This horn moves freely in a vertical plane when the larva is walking.

**Figure 26:**
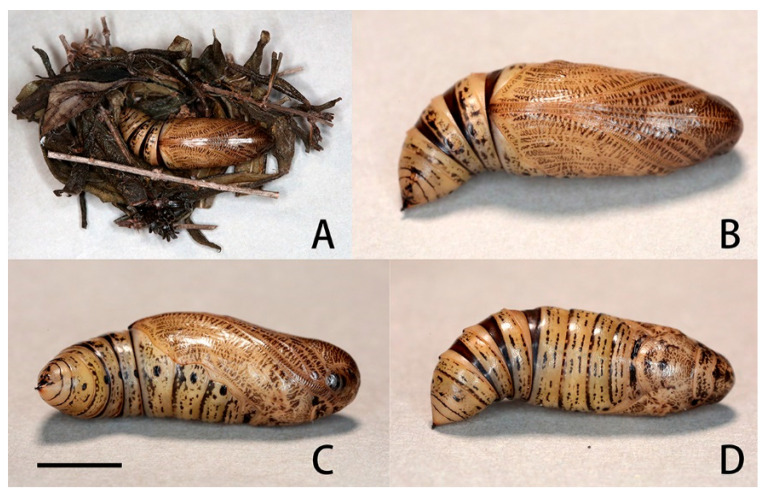
Pupal photo of *Neogurelca montana taihangensis* Xu & He **ssp. nov.** (**A**) Pupa and pupal chamber; (**B**) ventral view; (**C**) lateral view, scale bar = 10 mm; (**D**) dorsal view. Photos by Ming-Lei Bi. Scale bar = 10 mm.

Pupa (Figure 26): Body length 23 mm. Ground colour ochreous but translucent. Head, tongue, wing-cases, legs, antenna, and thorax with distinctly regular interrupted dark brown transverse bars, these more prominent dorsally. Intersegmental cuticle of movable segments 8, 9, and 10 deep chestnut. All spiracles and shaft of cremaster dark brown/black, with the spiracles lying on black patches. Head elongate but rounded in front; tongue-case not projecting and flush with the surface of the body; shoulders prominent. Antenna shorter than foreleg, and there is a well-developed coxal piece. Head with vertex. Thorax superficially, transversely, irregularly lined. Wing-case smooth, bulging ventrally. Abdomen smooth except for very superficial transverse folds and pits. There are very small erect hairs on the frons and around the spiracles. There is some sculpturing on segment 4 consisting of a small transverse subdorsal weal just behind the front margin, and a small pit above and behind the spiracle of 6. The spiracles of 9, 10, and 11 are situated on the posterior face of a ridge running round the front margins of those segments. Segment 9 has small, irregular, ante-spiracular ridges. Spiracle of 2 covered by a rounded lobe projecting from the front margin of 3, its front edge raised. Remaining spiracles broadly oval, edges of central slit raised. Cremaster very short, conical, ending in a short, stout, cylindrical shaft bearing about twelve very short-stemmed anchor-shaped hooklets.

Formed in leaf litter on the ground, a few thick strands of gummy silk used to hold the plant debris together to create a cocoon-like “chamber” but leaving part of the fold open frontad. Duration of pupa 14 d in autumn.

**Figure 27:**
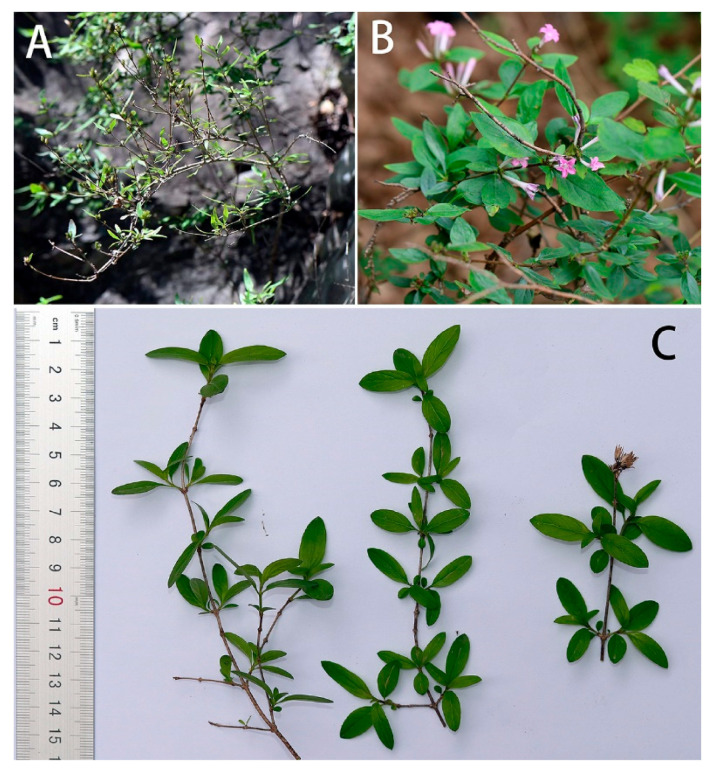
*Leptodermis oblonga*, Host plant of *Neogurelca montana taihangensis* Xu & He **ssp. nov.** in Beijing, China. (**A**) The shrub of *Leptodermis oblonga*, photo by Jia-Xuan Wang.; (**B**) tender branches, leaves, and flowers in the field; (**C**) tender branches and leaves with scale bar, photo by Yu-Chen Zhang.

Host plant (Figure 27): In 1914, Mell found a larva in ‘Tali’ [Dali, Yunnan], China, on the *Paederia foetida* L. He described the larva at the time as *Gurelca saturata* [11], which has now been synonymized with *N. montana montana*. In contrast, the larval host of *N. montana taihangensis* **ssp. nov**. in Beijing is *Leptodermis oblonga* Bunge (Rubiaceae: Paederieae). This plant is a small shrub growing up to 60–150 cm, which can be found on the rocky cliffs and soil slopes in the mountains of Beijing. The leaves are small and oval; the flowers are pink with long tubes. If the leaves are rubbed repeatedly, an odour such as *P. foetida* and other members of Paederieae is released.

As no host shift was tested on *N. montana taihangensis* **ssp. nov.** due to limited material available for rearing observation, acceptance for other plants in Rubiaceae (e.g., *Paederia foetida*, *Rubia cordifolia* L., and *Galium spurium* L.) is still unknown.

#### 3.3.2. *Neogurelca himachala*

**Figure 28:**
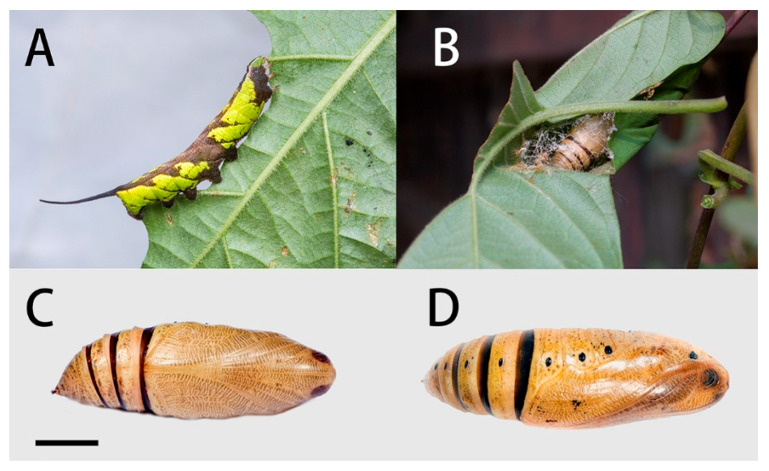
Life stages of *Neogurelca himachala*. (**A**) Final instar larva; (**B**) pupa in pupal chamber spun among leaves of host plant; (**C**) ventral view of pupa, scale bar = 10 mm.; (**D**) lateral view of pupa. All images by Ji-Bai He.

Final instar larva (Figure 28A): Body length 42–45 mm. Polychromatic: Dark brown, grey-brown or yellowish-green with brown markings. In the green form larva: Head green, nearly oval and held almost horizontally when at rest. A dark brown stripe separating face from cheek. Surface dull and set with short, curved, translucent hairs, each arising from a minute tubercle.

Body green, dull, smooth, and cylindrical with dark brown markings. A dark brown and yellow dorso-lateral stripe runs from segment 2 to the base of the horn, very narrow on 2 to 6, broader on the remaining segments. Segments 2 and 3 suffused with dark brown below the dorso-lateral stripe, and segment 4 with a smaller patch of dark brown not reaching the subdorsal stripe; the hind portion of segment 7–8 suffused with dark brown below the dorso-lateral stripe and also above it nearly to the dorsum. There are whitish oblique stripes, which are slightly defined on segments 5 to 7, missing on 8 and less defined on segments 9 to 11; all stop at the dorso-lateral stripe and are edged above to a greater or lesser extent by dark brown. The dark brown colour may spread on to the dorsum of segments 7 to 11 as a diamond-shaped mark on each segment. Ventral surface mainly brown on abdomen, less so on thorax. Spiracles white with a broad dark brown band across the middle.

Horn very long, smoky-black, fairly thick at base, tapering gently to beyond middle and then thickening gently, tip slightly bifid, each arm ending in two points, each of which bears a seta; the basal half of the horn straight and held normally horizontal, the distal half strongly upcurved; surface covered with very regularly placed, small black tubercles. This long horn moves freely in a vertical plane when the larva is walking.

Pupa (Figure 28C,D): Body length 23–25 mm; breadth 8 mm. Similar to *N. montana*, but ochreous in colour. The regular interrupted transverse bars on head, tongue, wing-case, legs, antenna and thorax of leaden colour, less developed than those of *N. montana*.

Formed in a folded leaf above (Figure 28B) or on the ground, this held together by a few thick strands of gummy silk but leaving part of the fold open frontad. As the larva shrinks it often forcibly ejects several jets of excess water through this opening.

**Figure 29:**
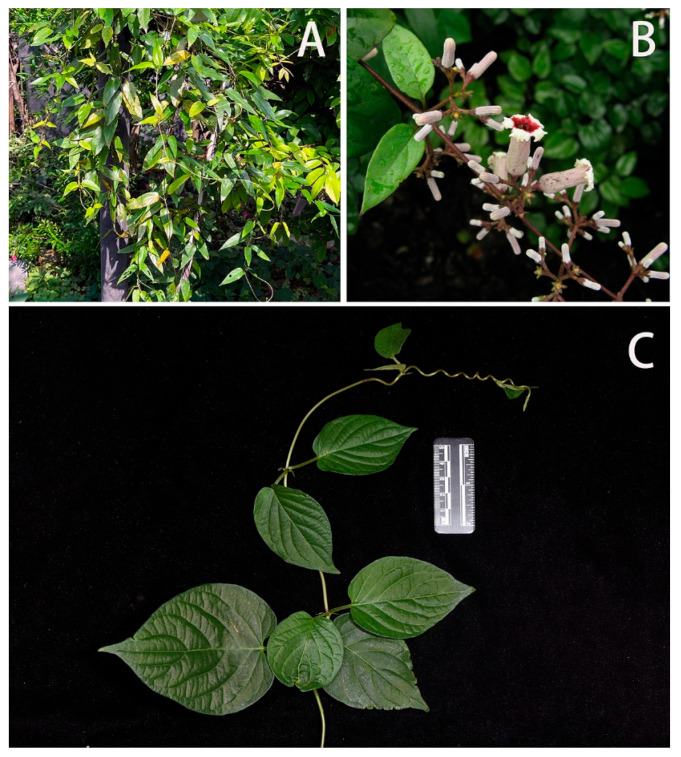
*Paederia foetida*, Host plant of *Neogurelca himachala* in China. (**A**) The vine of *Paederia foetida*, photo by Xin Lin; (**B**) tender branches, leaves, and flowers in the field, photo by Yu-Song Huang; (**C**) tender branches and leaves with scale bar, photo by Shi-Wei Guo.

Host Plant (Figure 29): In Shaanxi, Wuhan, Nanjing, Guangxi and Guangdong, larvae were found on *Paederia foetida* (Rubiaceae: Paederieae). The plant is an epiphytic vine that is very common in parks and shrubs in central and southern China. Leaves ovate, apex pointed; flowers white, bell-shaped. The whole plant exudes a strong foul smell.

## 4. Discussion

Our molecular, morphological, and life-history evidence all indicate that the *Neogurelca* from Beijing is *N. montana* rather than *N. himachala*. In the Bayesian tree, the Beijing population (BHS) was sister to *N. montana* (NMO) form Yunnan, then grouped with the clade of *N. himachala* (NHI) (Figure 2). The lateral abdominal hair tufts in Beijing *Neogurelca* well-developed, but that structure is absent in *N. himachala* (Figure 5C–F). In the male genitalia, the phallus of Beijing *Neogurelca* has a transverse apical process, while in *N. himachala* it is scimitar-shaped. The patterns of larvae and pupae are obviously different in Beijing *Neogurelca* and *N. himachala*, with the former closer to the *montana* larvae recorded by Mell [11] in Yunnan. Hence, *N. montana* is distributed in Beijing and Hebei (Taihang Mountain area) as well as Sichuan, Yunnan, and Tibet. *N. himachala* is distributed from eastern China, southern China to the Himalayas, and also found in Taiwan Island, through the Ryukyu Islands to the south of the Korean Peninsula.

Our study showed that *N. montana taihangensis* **ssp. nov.** and the nominotypical subspecies are differentiated. The K2P genetic distance of the two is very small (0.9%), much lower than those between congeneric species (see ‘molecular species delimitation’ above). However, the two populations form two monophyletic groups in the Bayesian tree (Figure 2), indicating their distinct phylogenetic characters. Morphological analysis of genital structures as well as body and wing patterns are consistent with the molecular evidence (Figure 3, Figure 4, Figure 5, Figure 6, Figure 7, Figure 8, Figure 9, Figure 10 and Figure 11). Therefore, we assign subspecific status to *N. montana taihangensis* **ssp. nov.** Although there is a geographical gap between Taihang Mountain and the Hengduan Mountains in our sampling effort, our four-year survey did not collect any specimen in the potential distribution range of *N. montana* between the two known distributional areas, e.g., Mt. Qinling. Future research must address this point to form a better understanding of the subspecies divergence, diversity, and distribution pattern when samples from the gap area become available.

Female genitalia have been long neglected because it was considered they lack rapid and divergent evolution in their morphology [39]. However, recent studies have shown that both male and female genital morphology have coevolved in several groups of animals producing complex and diverse structures that play a broad spectrum of functions [40,41,42]. Our morphological analysis supports the above conclusion. The female genitalia of *N. montana* has a heavily sclerotized and twisted colliculum, while in *N. himachala*, it is only lightly sclerotized, short, and straight. Like in male genitalia, such differences can help distinguish *N. montana* from Beijing and *N. himachala*. The comparison of female genitalia in other *Neogurelca* species also demonstrated similar differences, suggesting that collecting female specimens and morphological comparison of female genitalia should be accorded appropriate attention in future taxonomic studies.

**Figure 30:**
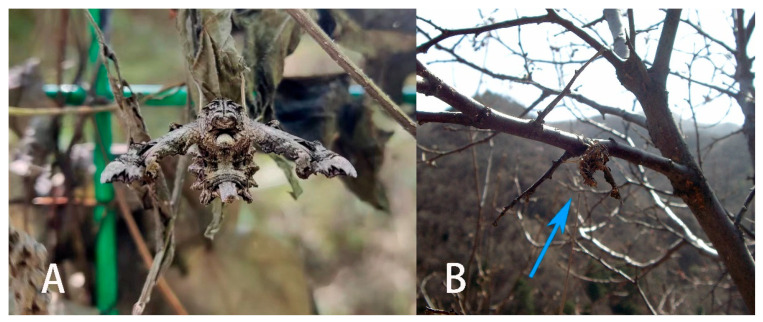
Live adults of *Neogurelca montana taihangensis* **ssp. nov.** and habitat. (**A**) A female adult on a dead leaf, photo by Nan Yang; (**B**) adult (blue arrow) on a branch in early spring in Beijing, photo by Nan Yang.

Masquerade mimicry as leaves or sticks is frequently found throughout the animal kingdom, such as in the Malayan leaf frog *Megophrys nasuta*, stick insects (Phylliidae), and oakleaf butterflies (Nymphalidae) [43,44,45]. This morphological blending with the environment is more obvious in *N. montana*, which has a darker body colour and a more stick-like body than in other *Neogurelca* species (Figure 30A,B). In addition, the special arrangement of the scales on the thorax and abdomen could also help *N. montana* mimic the dead leaves or branches in its habitat (Figure 30A) [46]. Here, we speculate that such ecological adaptation traits are worthy of taxonomic attention, as they may imply different evolutionary contexts, i.e., different habitats. Therefore, it is important to ensure the quality of specimens during collection and preservation to avoid abrasion or loss of scales and hairs bearing valuable morphological information.

## Figures and Tables

**Table 1:** Sampling information and BOLD SampleID/GenBank accession numbers of *Neogurelca* and outgroups used in this study. The taxon names follow current taxonomy, mentioned above.

Taxon (Sample Code)	Locality	Collecting Date	GenBank No.	Bold ID
*N. himachala* (NHI02)	Wuhan, Hubei, China	2018-XI	OR235183	
*N. himachala* (NHI03)	Wuhan, Hubei, China	2018-XI	OR235184	
*N. himachala* (NHI04)	Wuhan, Hubei, China	2018-XI	OR235185	
*N. himachala* (NHI06)	Baotianman, Henan, China	2021-IX	OR235186	
*N. himachala* (NHI10)	Niubeiliang, Shaanxi, China	2017-VII	OR235187	
*N. himachala*	Zhejiang, China	2003-I		IJK-04-0004
*N. hyas* (NHA01)	Lingao, Hainan, China	2023-VI	OR235191	
*N. hyas* (NHA02)	Lingao, Hainan, China	2023-VI	OR235192	
*N. hyas*	Lomblen Island, Indonesia	2006-XI		BC-Mel 0555
*N. hyas*	Leyte Islands, Philippines	2003-VIII		BC-Hax3980
*N. montana montana* (NMO01)	Shangri-La, Yunnan, China	2017-VIII	OR235188	
*N. montana montana* (NMO02)	Cangshan, Dali, Yunnan, China	2020-XI	OR235189	
*N. montana montana* (NMO03)	BingZhongluo, Yunnan, China	2021-VII	OR235190	
*N. montana taihangensis* **ssp. nov.** (BHS01)	Baihuashan, Beijing, China	2018-X	OR235181	
*N. montana taihangensis* **ssp. nov.** (BHS04)	Yunmengshan, Beijing, China	2018-VII	OR235182	
*Sphingonaepiopsis pumilio*	Sumatra, Indonesia	2004-XI		BC-Mel 0221
*Sphingonaepiopsis gorgoniades*	Ukraine	2005-I		IJK-05-0003

**Table 2:** Kimura two-parameter (K2P) distances (in percentages) between all taxa of the genus *Neogurelca* calculated from the DNA barcode sequences (*cox1*), with species identified as in the Bayesian phylogenetic tree in Figure 2.

	1	2	3	4
1. *Neogurelca montana taihangensis* **ssp. nov.**				
2. *Neogurelca montana montana*	0.9			
3. *Neogurelca himachala*	5.9	5.6		
4. *Neogurelca hyas*	9.2	8.7	10.0	

**Table 3:** Monophylizer assessment of the *Neogurelca* species and subspecies used in this study.

Taxon	Assessment	Tangles
1. *Neogurelca montana taihangensis* **ssp. nov.**	monophyletic	—
2. *Neogurelca montana montana*	monophyletic	—
3a. *Neogurelca himachala*	monophyletic	—
3b. *Neogurelca hyas*	monophyletic	—

## Data Availability

The data are openly available in GenBank at https://www.ncbi.nlm.nih.gov/genbank/ and BOLD SYSTEMS https://v4.boldsystems.org/ (accessed on 11 October 2023). The list of investigated species and their GenBank accession numbers or BOLD sampleIDs are given in Table 1.

## Appendix A. List of *Neogurelca* Specimens Examined

Names of institutions in which specimens are deposited are listed after private collections and are abbreviated as follows: CQL—collection of Chang-Qiu Liu (Guilin, China); ZBX—collection of Zhen-Bang Xu (Guilin, China); TL—collection of Tao Li (Beijing, China); ZHJ—collection of Zhuo-Heng Jiang; NHMUK—collections of the Natural History Museum (London, United Kingdom); KIZ—collections of the Kunming Institute of Zoology, Chinese Academy of Science (Kunming, Yunnan, China); IOZ—collections of the National Animal Collection Resource Center, Institute of Zoology, Chinese Academy of Sciences.

***Neogurelca montana montana*** **(Rothschild & Jordan, 1915)**

**SYNTYPES:** 4♂♂, Thibet [Tibet], China, without more definite locality and date information, Le Moult *leg*. [NHMUK 014200047, NHMUK 014200048, NHMUK 014200049 and NHMUK 014200050].

**Additional specimens:** ♂, Shangri-La Alpine Botanical Garden (3300 m), Diqing, Yunnan, China, 2017-VIII, Chang-Qiu Liu *leg*. [ZBX]; ♂, Bingzhongluo, Nujiang, Yunnan, China, 2021-VII-26 Si-Yao Huang *leg*. [KIZ 0132781]; ♀, Cangshan (2200 m), Dali, Yunnan, China, 2020-XI-9, Lan-Fang Zhao *leg*. [ZBX]; ♀, Nyingchi (2400 m), Tibet, China, 2021-VI-4, Guo-Cong Huang *leg*. [ZHJ]; ♀, Cangshan (2350 m), Dali, Yunnan, China, 2021-X-14, Wei Zhang *leg*. [ZHJ].

***Neogurelca montana taihangensis*** **Xu & He ssp.nov**

**HOLOTYPE:** ♂, Huangshandian village (130 m), Fangshan District, Beijing, China, 2022-IX-13, Zhen-Bang Xu *leg*. [KIZ 0132780]. **PARATYPES**: ♂, Beijing Baihuashan National Nature Reserve (1400 m), Mentougou District, Beijing, China, 2018-X, Nan Yang *leg*. [ZBX]; ♂, Xiaolongmen Forest Farm (1, 100 m), Mentougou District, Beijing, China, 2021-VIII, Hong-Lin Zhu *leg*. [ZBX]; ♂, same locality, 2022-VI-5, Hong-Lin Zhu *leg*. [ZBX]; ♂, China National Botanical Garden, Haidian District, Beijing, China, 2022-X-1, Yu-Chen Zhang *leg*. [ZBX]; ♂, Yue’an mountain forestry centre of Beijing University of Agriculture (800 m), Sidaohe Village, Baoshan Town, Huairou District, Beijing, China, 2022-VIII-15, Tao Li *leg*. [IOZ 221490]; ♀, Beijing Baihuashan National Nature Reserve (1400 m), Mentougou District, Beijing, China, 2013-VI, Nan Yang *leg*. [ZBX]; ♀, Yunmengshan National Forest Park, Minyun District, Beijing, China, 2018-VII, Chen Wang *leg*. [ZBX]; ♀, China National Botanical Garden, Haidian District, Beijing, China, 2022-X-1, Yu-Chen Zhang *leg*. [ZBX].

***Neogurelca himachala*** **(Butler, 1876)**

**LECTOTYPE:** ♂, N.E. Himalaya, Farr *leg*. [NHMUK]. **SYNTYPES:** ♀, N. China, Shanghai [Shanghai], [NHMUK]; ♂, N.W. India, [NHMUK].

**Additional specimens:** 2♂♂, Wuhan, Hubei, China, 2018-XI, Ji-Bai He *leg*. [ZBX]; ♀, same collecting data, [ZBX]; ♂, Wuhan, Hubei, China, 2018-V, Ji-Bai He *leg*. [ZBX]; ♂, Wuhan, Hubei, China, 2018-V, Ji-Bai He *leg*. [ZBX]; ♀, Xinyang, Henan, China, 2022-VI, Chong-Lin Wang *leg*. [ZBX]; ♂, Baotianman, Nanyang, Henan, China, 2021-IX, Chong-Lin Wang *leg*. [ZBX]; ♀, Niubeiliang, Shangluo, Shaanxi, China, 2017-VII, Chang-Qiu Liu *leg*. [CQL]; ♂, Niubeiliang, Shangluo, Shaanxi, China, 2017-VII, Chang-Qiu Liu *leg*. [CQL]; ♂, Guilin, Guangxi, China, 2021-X, Chang-Qiu Liu *leg*. [ZBX]; ♂, Nanjing, Jiangsu, China, 2021-VIII, Dong Wang *leg*. [ZBX]; ♀, Hangzhou, Zhejiang, China, 2023-VI, Zhuo-Heng Jiang *leg*. [ZHJ]; ♀, Molangshan (1170 m), Yuxi, Yunnan, China, 2017-VIII, Zhuo-Heng Jiang *leg*. [ZHJ]; ♂, Funing, Yunnan, China, 1973-VIII, Yun-Xing Gan *leg*. [KIZ 0045664].

***Neogurelca masuriensis*** **(Butler, 1875)**

**SYNTYPES:** 3♂♂, N.W. Himalaya, [India, Uttar Pradesh,] Masuri [Mussoorie] [NHMUK]; 3♀♀, same collecting data, [NHMUK].

***Neogurelca hyas*** **(1856)**

**LECTOTYPE:** Java [Indonesia], [NHMUK]. **PARALECTOTYPES:** ♀, no collecting data, [NHMUK]; ♀, Silhet [Bangladesh, Sylhet], [NHMUK]; ♂, Hong Kong, [NHMUK].

**Additional specimens:** 2♂♂, Lingao, Hainan, China, 2023-VI, local catcher *leg*. [ZBX]; ♂, Guangzhou, Guangdong, China, 2022-IV, local catcher *leg*. [ZHJ]; ♂, same collecting data, [ZBX].
